# Effect of atelectasis changes on tissue mass and dose during lung radiotherapy

**DOI:** 10.1118/1.4965807

**Published:** 2016-10-24

**Authors:** Christopher L. Guy, Elisabeth Weiss, Nuzhat Jan, Leonid B. Reshko, Gary E. Christensen, Geoffrey D. Hugo

**Affiliations:** Department of Radiation Oncology, Virginia Commonwealth University, Richmond, Virginia 23298; Department of Electrical and Computer Engineering and Department of Radiation Oncology, University of Iowa, Iowa City, Iowa 52242; Department of Radiation Oncology, Virginia Commonwealth University, Richmond, Virginia 23298

**Keywords:** lung cancer, atelectasis, image analysis, lung, tissue characterization

## Abstract

**Purpose:**

To characterize mass and density changes of lung parenchyma in non-small cell lung cancer (NSCLC) patients following midtreatment resolution of atelectasis and to quantify the impact this large geometric change has on normal tissue dose.

**Methods:**

Baseline and midtreatment CT images and contours were obtained for 18 NSCLC patients with atelectasis. Patients were classified based on atelectasis volume reduction between the two scans as having either full, partial, or no resolution. Relative mass and density changes from baseline to midtreatment were calculated based on voxel intensity and volume for each lung lobe. Patients also had clinical treatment plans available which were used to assess changes in normal tissue dose constraints from baseline to midtreatment. The midtreatment image was rigidly aligned with the baseline scan in two ways: (1) bony anatomy and (2) carina. Treatment parameters (beam apertures, weights, angles, monitor units, etc.) were transferred to each image. Then, dose was recalculated. Typical IMRT dose constraints were evaluated on all images, and the changes from baseline to each midtreatment image were investigated.

**Results:**

Atelectatic lobes experienced mean (stdev) mass changes of −2.8% (36.6%), −24.4% (33.0%), and −9.2% (17.5%) and density changes of −66.0% (6.4%), −25.6% (13.6%), and −17.0% (21.1%) for full, partial, and no resolution, respectively. Means (stdev) of dose changes to spinal cord *D*_max_, esophagus *D*_mean_, and lungs *D*_mean_ were 0.67 (2.99), 0.99 (2.69), and 0.50 Gy (2.05 Gy), respectively, for bone alignment and 0.14 (1.80), 0.77 (2.95), and 0.06 Gy (1.71 Gy) for carina alignment. Dose increases with bone alignment up to 10.93, 7.92, and 5.69 Gy were found for maximum spinal cord, mean esophagus, and mean lung doses, respectively, with carina alignment yielding similar values. 44% and 22% of patients had at least one metric change by at least 5 Gy (dose metrics) or 5% (volume metrics) for bone and carina alignments, respectively. Investigation of GTV coverage showed mean (stdev) changes in *V_Rx_*, *D*_max_, and *D*_min_ of −5.5% (13.5%), 2.5% (4.2%), and 0.8% (8.9%), respectively, for bone alignment with similar results for carina alignment.

**Conclusions:**

Resolution of atelectasis caused mass and density decreases, on average, and introduced substantial changes in normal tissue dose metrics in a subset of the patient cohort.

## 1. INTRODUCTION

Obstructive lobar atelectasis, the collapse of lung tissue due to restricted airflow, commonly occurs in nonsmall cell lung cancer (NSCLC) patients with centrally located tumors.^1^ Initial atelectasis presentation rates for patients undergoing external beam radiotherapy have been reported to range between 10% and 40%.^2–5^ During the course of radiotherapy, tumor regression or progression can cause changes in atelectasis, either resolution or expansion. Studies investigating anatomical variations during treatment found atelectasis changes in 10% to 30% of all NSCLC patients.^3,5,6^

Resolution of atelectasis, in the case of full reaeration of whole lobe collapse, appears in computed tomography (CT) scans as a change from a uniform, high-intensity consolidated volume to a larger, lower-intensity region of normal parenchyma. This can produce large geometric changes in treatment anatomy which can cause baseline shifts in tumor position.^3,6^ These large geometric changes impact dose to the target and critical structures and cannot be handled by treatment margins, instead requiring plan adaptation.^3^ Anatomical variations have been shown to have a greater impact on target dose than either respiratory motion or baseline shifts (e.g., setup errors), highlighting the potential need for adaptive radiotherapy in patients with atelectasis changes.^7^

Little has been reported about the characteristics of atelectasis changes during radiotherapy, partly due to a lack of diagnostic-quality imaging during treatment. The aforementioned studies relied on followup CTs taken months or years after treatment^2,4^ or cone-beam CT scans which have relatively poor contrast resolution and electron density inaccuracies^3,5,6^ which make clear identification of the atelectatic regions challenging. The purpose of this study is to quantitatively characterize mass and density changes of obstructive lobar atelectasis during treatment in NSCLC patients using weekly helical CTs and to investigate the dosimetric impact of such changes on normal tissue structures in lieu of adaptive replanning. It is anticipated that studying these mass changes will provide a better understanding of how atelectasis resolution impacts dose calculation and image registration in a sizable subset of NSCLC patients.

## 2. MATERIALS AND METHODS

### 2.A. Data acquisition

Pairs of baseline and midtreatment CT scans for eighteen patients were acquired on a CT simulator (Philips Brilliance Big Bore, Fitchburg, WI) under IRB-approved protocols. The baseline scan was taken at or near the time of simulation, and the midtreatment scan was taken during the course of radiotherapy, with a mean (stdev) of 46 (12) days between the two scans. Five patients (28%) underwent breath hold scans, while the remainder (72%) had free breathing 4DCT acquisitions. Four of the five breath hold patients also had repeat scans taken during each weekly session, making available three images at all time points. For all but four patients, the planning CT was used as the baseline scan. The remaining four patients had 4DCT planning images, but breath hold midtreatment imaging. For consistency, a breath hold image acquired close to the time of planning CT acquisition was used as the baseline scan. The 50% phase (end-of-expiration) of each 4D image was selected for use in this study as it is considered to have minimum tissue motion and therefore likely to have the least sorting and motion artifacts. Both images of every pair were of the same scan type. Voxel size ranged from 1.17 × 1.17 × 2 mm^3^ to 1.37 × 1.37 × 3 mm^3^. Images of each pair had identical voxel size for all but two patients.

Tumor staging varied across patients between IB (5%), IIB (5%), IIIA (50%), and IIIB (40%) with a mean (stdev) baseline tumor volume of 109.6 (89.2) ml across patients. Tumor locations were as follows: left upper lobe (LUL) (17%), left lower lobe (LLL) (22%), right upper lobe (RUL) (22%), right middle lobe (RML) (6%), and right lower lobe (RLL) (50%). Treatment technique varied between 3D conformal and IMRT for the study cohort. Dose was delivered with conventional fractionation of 1.8–2.0 Gy per fraction, with a mean (stdev) prescription dose of 63.2 (5.0) Gy. Initial collapse type was scored based on whether the whole lobe (WL) (56%) or part of the lobe (PL) (44%), aside from tumor, was considered to be atelectatic. Patients had a mean (stdev) baseline atelectasis volume of 232.42 (181.55) ml. 17% of patients experienced collapse in the LUL, 17% in the LLL, 17% in the RUL, 11% in the RML, and 50% in the RLL. For some patients, the tumor and/or atelectasis was present in multiple lung lobes.

### 2.B. Atelectasis classification

Subjects were classified according to resolution of atelectatic tissue from baseline to followup. Classification was based on the change in atelectasis volume: greater than 80% volume reduction was labeled as full (22%), between 80% and 20% volume reduction was labeled as partial (50%), and a decrease in volume less than either 20% or 15 ml was labeled as no resolution (28%).

### 2.C. Lobe segmentation

Accurate delineation of the boundary of atelectasis can be challenging, particularly in cases where the atelectasis may be partially resolved. In addition to contouring the atelectasis directly, individual lung lobes were delineated to reduce the impact of delineation error since the lobe boundaries are generally more clearly defined. All fives lobes (right upper, right middle, right lower, left upper, and left lower) were delineated by individuals (NJ, LR) trained by an experienced radiation oncologist (EW) using a commercial radiation oncology software suite (MIM Maestro v6.6.4, Cleveland, OH). All delineations were reviewed by the same oncologist for accuracy and consistency. The tumor, atelectasis, and all five lung lobes were contoured in each image. For patients with repeat scans during each weekly session, the contours for each scan were drawn independently.

**FIG. 1.**
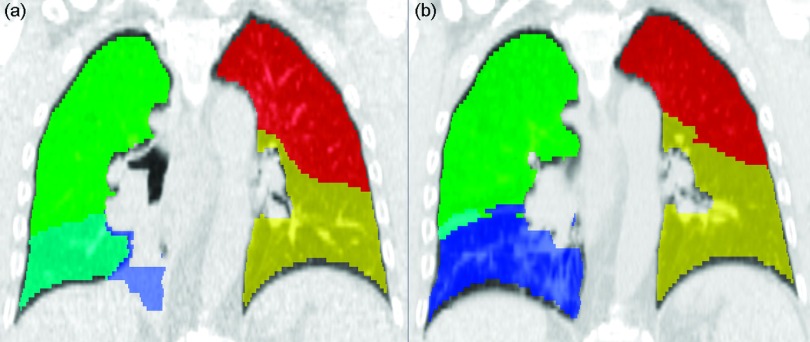
Lobe segmentation example. Lobe labels for patient 6 are shown for (a) baseline, where the lower right lobe (blue) is fully collapsed, and (b) followup lobes, where the right lower lobe has fully reaerated revealing healthy lung parenchyma and vessel structures. The right lower lobe expands to fill the anterior pleural cavity, pushing the right middle lobe (cyan) posterior to the shown coronal slice. Back-to-back 3D erosions of 1 voxel from the exterior of the lungs were performed to exclude extra-pleural tissue. The gross tumor volume has also been removed from the lobe masks.

Contours were converted to binary masks from which the tumor was removed, as tumor regression is known to occur and is not the focus of the study. To remove nonlung tissue inadvertently included in the lobe delineations, a combined lung binary mask was created which was then eroded by 1 voxel in all dimensions a total of two times. Eroded lobe masks were obtained by taking the union of the original lobe mask and the eroded combined lung mask, effectively eroding the individual lobe from only the exterior of the lung. An example of eroded lobe masks for one subject is shown in Fig. 1.

### 2.D. Mass and density calculation

Images were preprocessed to enable mass and density calculation. Variability in scanner performance over time was removed by linearly calibrating the images according to the average intensities of air outside the body and blood in the descending aorta, as described by Staring *et al.*^8^ The voxel values of the calibrated images were then in units of relative physical density, with air at approximately 0 mg/cc and blood at about 1050 mg/cc. Relative density was calculated by averaging calibrated voxel values in the region of interest, while relative mass was obtained by summing the product of voxel intensity and voxel volume throughout the region of interest. Relative mass and density change from baseline to midtreatment were calculated for all lobes of each patient. For the four patients with repeat weekly scans, each scan at the baseline time point was paired with a scan of the followup time point forming three image pairs per patient for which mass and density changes were calculated.

### 2.E. Treatment planning

Clinical treatment plans created with Philips pinnacle treatment planning system (TPS) (Philips Radiation Oncology Systems, Fitchburg, WI) were available for all patients. To investigate the dosimetric impact of atelectasis reaeration, dose was calculated on both the baseline and midtreatment scans using the exact clinical treatment parameters (beam angles, beam weights, MLC segments, etc.). In cases where the baseline image was not the clinical planning CT, the treatment plan was first transferred to the baseline scan via rigid registration of bony anatomy using the image fusion tools of MIM.

#### 2.E.1. Midtreatment alignment

Two methods were used to align the baseline plan to the midtreatment image in order to simulate different methods of daily patient setup. In method one (bone-aligned), the midtreatment image was rigidly registered to the baseline scan based on bony anatomy, and the resulting fusion was adjusted with box-based alignment of a region covering the sternum and spine. In the second method (carina-aligned), the default whole-body fusion was performed again, but with subsequent manual translational adjustment to align the carina region, mimicking carina-based volumetric image-guided setup.

#### 2.E.2. Dosimetric evaluation

Using the clinical beams (weights, apertures, and angles) and monitor units, dose was calculated and evaluated for the baseline plan, the bony-aligned midtreatment plan, and the carina-aligned midtreatment plan. It is important to note that this method assumes atelectasis resolution occurs before the first treatment fraction and represents a worst-case estimate of dose changes. To quantify the dosimetric differences in plan quality, a combination of RTOG 0617 and in-house normal tissue constraints was evaluated (see Table I for the complete list). Lungs were defined in three different ways: all lung tissue, lung tissue without gross tumor volume (GTV), and lung tissue without clinical target volume (CTV) in order to manage the effect of tumor regression on dose change assessment. The lung and GTV delineations were made on each scan separately. The CTV was rigidly transferred from the clinical planning image to baseline and followup scans after image alignment was performed. Inclusion of lungs and tumor together is expected to minimize the influence of tumor regression on dose changes, while removal of the GTV and CTV from the lung is expected to incorporate this effect.

**TABLE I.** Dose constraint metric changes.

			Bone aligned				Carina aligned			
Structure	Metric	Units	Mean	Stdev	Min	Max	Mean	Stdev	Min	Max
Spinal cord	*D*_max_	Gy	0.67	2.99	−2.78	10.93	0.14	1.80	−2.94	4.29
Esophagus	*D*_mean_	Gy	0.99	2.69	−3.72	7.92	0.77	2.95	−4.56	7.07
Heart	*V*_40_	% Vol.	1.64	5.64	−16.15	9.72	1.59	3.62	−10.51	6.28
Heart	*V*_60_	% Vol.	0.96	2.14	−2.87	4.38	0.83	1.59	−3.58	2.64
Lungs	*D*_mean_	Gy	0.50	2.05	−2.89	5.69	0.06	1.71	−3.35	4.56
Lungs	*V*_20_	% Vol.	1.17	3.42	−3.22	11.31	0.28	2.56	−4.15	6.55
Lungs	*V*_30_	% Vol.	0.87	2.86	−3.59	6.44	0.19	2.55	−4.22	6.97
Lungs-CTV	*D*_mean_	Gy	0.61	1.72	−1.80	5.61	0.18	1.31	−1.87	4.02
Lungs-CTV	*V*_20_	% Vol.	1.40	3.14	−2.87	11.43	0.49	2.15	−2.99	5.86
Lungs-CTV	*V*_30_	% Vol.	1.11	2.41	−2.66	6.42	0.41	2.04	−2.94	6.17
Lungs-GTV	*D*_mean_	Gy	0.99	2.23	−1.70	5.94	0.55	2.09	−1.83	7.39
Lungs-GTV	*V*_20_	% Vol.	1.90	3.59	−2.72	11.35	1.00	3.09	−2.83	10.56
Lungs-GTV	*V*_30_	% Vol.	1.65	3.24	−2.50	9.35	0.97	3.19	−2.77	11.34

Note: Shown are changes in dose metrics from baseline to followup for bone and carina followup alignments. Mean and standard deviation were taken over all subjects. Also shown are the largest increases and decreases in metric values experienced by any subject.

### 2.F. Analysis

Analyses were performed with R 3.2.1. To determine if mass and density changes differed between healthy lobes (contralateral and pathology-free ipsilateral) and atelectatic lobes, a Wilcoxon rank sum test was used. An F-test was used to determine if variance in dose changes was significantly different between patients showing no resolution and those experiencing partial or full resolution. All tests used a 0.95 confidence level and were unpaired and two-sided. Additionally, mean and standard deviation of all changes were calculated.

## 3. RESULTS

### 3.A. Mass change

**FIG. 2.**
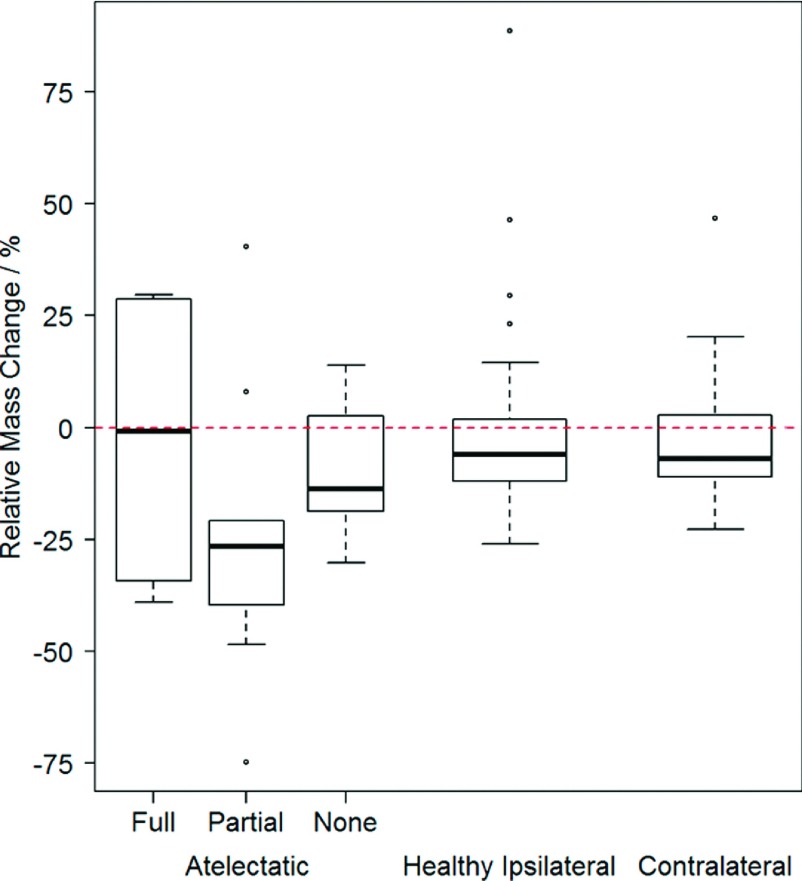
Box plots of percent change in relative mass from baseline to followup are shown for atelectatic lobes (left), healthy ipsilateral lobes (center), and contralateral lobes (right). Lobes containing atelectasis are subdivided by resolution type.

Changes in lobe mass from baseline to followup, as a percentage of the baseline value, are shown in Fig. 2. The mean (stdev) of mass change for all healthy contralateral (*n* = 41) and healthy ipsilateral (*n* = 29) lobes was −3.7% (12.2%) and 0.0% (23.0%), respectively. There was no significant difference between the two healthy lobe groups (*p* = 1). For atelectatic lobes, changes of −2.8% (36.6%), −24.4% (33.0%), and −9.2% (17.5%) were found for full resolution (*n* = 4), partial resolution (*n* = 9), and no resolution (*n* = 5) cases, respectively. Mass change was not significantly different from healthy lobes for full resolution (*p* = 0.9) or no resolution (*p* = 0.4) lobes. However, partial resolution mass change showed significant difference from that of healthy lobes (*p* = 0.005). For patients with multiple scans per session, intrapatient standard deviation of mass change taken across the three image pairs was 4.7% for healthy lobes and 3.5% for atelectatic lobes, on average.

### 3.B. Density change

**FIG. 3.**
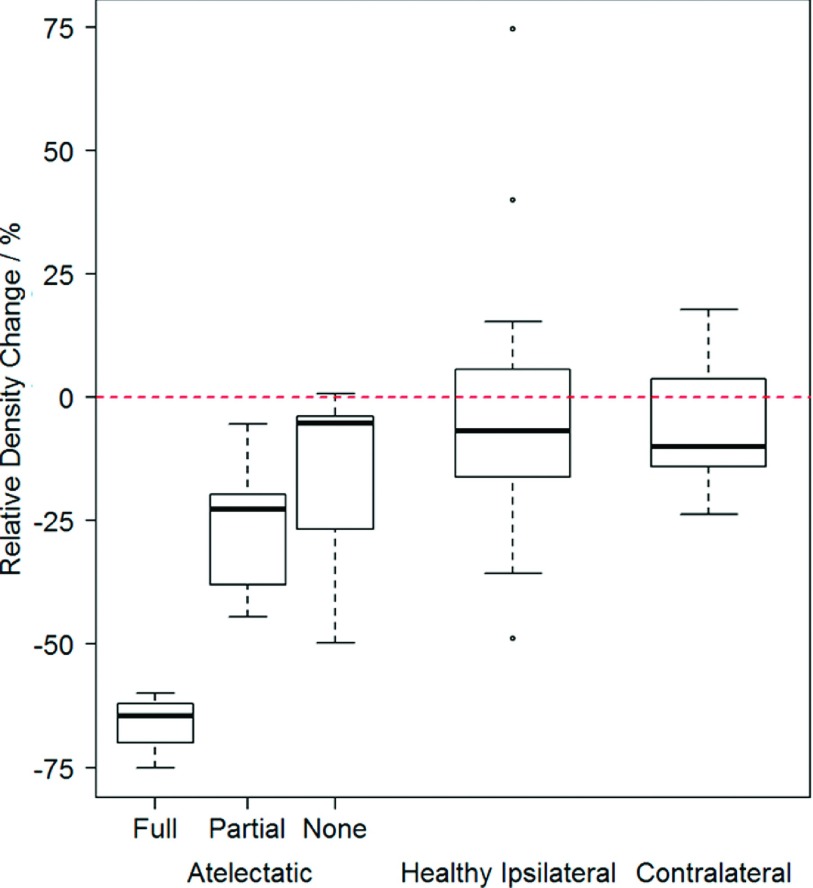
Box plots of percent change in relative density from baseline to followup are shown for atelectatic lobes (left), healthy ipsilateral lobes (center), and contralateral lobes (right). Lobes containing atelectasis are subdivided by resolution type.

Changes in lobe density are shown in Fig. 3. Lobes containing atelectasis experienced changes in density, from baseline to followup, of −66.0% (6.5%), −25.6% (13.6%), and −17.0% (21.1%) for full, partial, and no resolution, respectively. Density changes for healthy ipsilateral and contralateral lobes were −3.5% (23.3%) and −5.2% (12.0%), respectively. There was no significant difference in density change between healthy ipsilateral and contralateral (*p*  =  0.9) or between no resolution and healthy lobes (*p* = 0.3). Significant differences were present between full resolution and healthy lobes (*p* = 0.0008) and partial resolution and healthy lobes (*p* = 0.0006). Among the four patients with multiple image pairs, average intrapatient standard deviation of density change was 3.5% for healthy lobes and 0.6% for atelectatic lobes.

### 3.C. Volume change

**FIG. 4.**
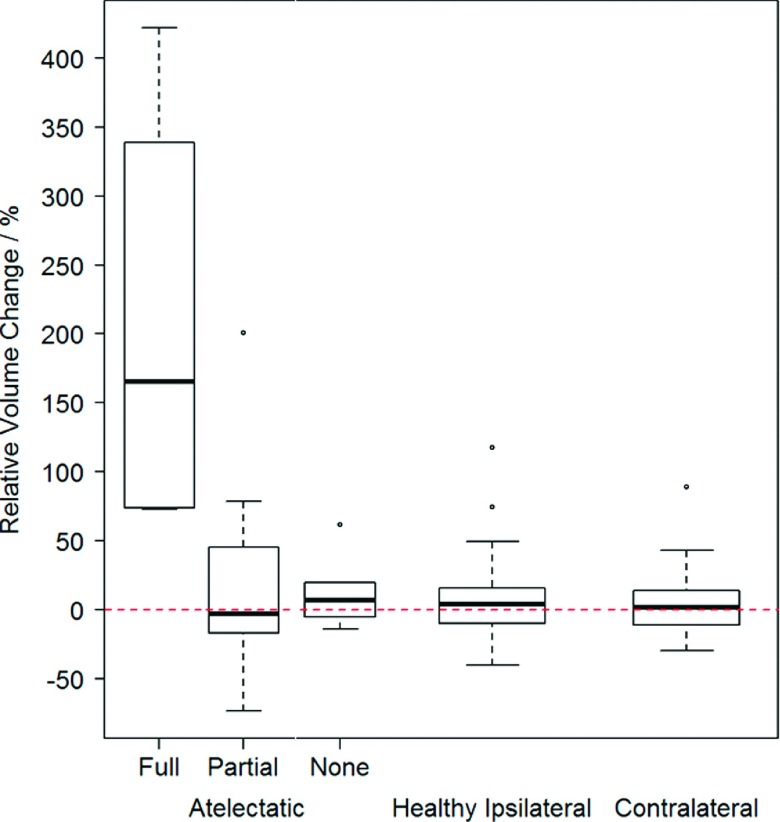
Box plots of percent change in relative volume from baseline to followup are shown for atelectatic lobes (left), healthy ipsilateral lobes (center), and contralateral lobes (right). Lobes containing atelectasis are subdivided by resolution type.

Changes in lobe volume are shown in Fig. 4. Lobes containing atelectasis experienced changes in volume, from baseline to followup, of +206% (167%), +22.5% (79.1%), and +14.1% (29.5%) for full, partial, and no resolution, respectively. Volume changes for healthy ipsilateral and contralateral lobes were +8.6% (33.1%) and +3.7% (21.6%), respectively. There was no significant difference in volume change between healthy ipsilateral and contralateral (*p* = 0.8), between no resolution and healthy lobes (*p* = 0.5), or between partial resolution and healthy lobes (*p* = 0.8). A significant difference in mean volume change was present between full resolution and healthy lobes (*p* = 0.001).

### 3.D. Organ at risk dose

**FIG. 5.**
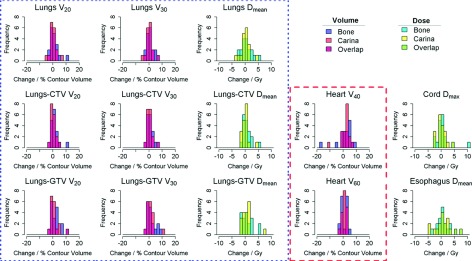
Histograms of change in dose and volume metrics from baseline to followup are shown for bone and carina alignment for all evaluated dose constraints. Bin widths were set to 1 Gy for dose metrics and 2% for volume constraints. The red dashed box surrounds heart constraints, while the blue dotted box encompasses lung constraints.

Dose and volume changes from baseline to followup were analyzed across all patients using available treatment plans. Changes in dose constraint metrics are shown in Table I. While mean dose changes were less than the typical dose per fraction of 2 Gy, very large dose and volume changes occurred for a subset of patients. In particular, dose increases from baseline to followup of up to 10.93, 7.92, and 5.69 Gy were found for maximum spinal cord dose, mean esophagus dose, and mean lung-GTV dose, respectively, when the subject was aligned via bone. Maximum changes were slightly reduced for carina alignment. Histograms of changes found for each dose metric are shown in Fig. 5 for both followup alignments. Across all patients, the percentage of changes exceeding 1 Gy/1%, 2 Gy/2%, 5 Gy/5%, and 10 Gy/10% were 63%, 38%, 12%, and 2%, respectively, for bone alignment and 62%, 28%, 5%, and 1% for carina alignment. The number of patients with at least one change larger than 5 Gy/5% was 44% for bone alignment and 22% for carina alignment.

Change in constraint metric value relative to baseline value was assessed in relation to atelectasis resolution type. The mean (stdev) relative changes in metrics across all patients for full, partial, and no resolution were 7.9% (18.6%), 18.6% (63.2%), and 1.9% (11.5%) for bone alignment, respectively. Deviations were similar for carina alignment with mean (stdev) relative changes of 13.0% (19.1%), 5.5% (27.8%), and −1.0% (10.6%) for full, partial, and no resolution, respectively. There was a significant difference between variances of changes between patients experiencing some degree of resolution (full or partial) and those showing no change in atelectasis for both alignments (*p* ≪ 0.05).

Table II lists dose constraints investigated along with the number of plans meeting the constraints for each plan type. Also shown is the number of metrics changing from being met in baseline to unmet in followup and vice-versa. For bone-aligned followup plans, four subjects had at least one OAR metric improve, e.g., change from being unmet in baseline to being met in followup, and seven subjects had at least one metric worsen. With carina alignment, four subjects had at least one metric improve and six subjects had at least one metric worsen, though some patients differed between the two groups. It should be noted that lungs were defined to include atelectasis in this study, whereas lung delineations of the original clinical treatment plans excluded atelectatic tissue, explaining the lack of baseline plans meeting lung constraints.

**TABLE II.** Dose constraints.

			No. of plans meeting constraint			No. improved		No. worsened	
Structure	Metric	Limit	BL	FU_Bone_	FU_Carina_	FU_Bone_	FU_Carina_	FU_Bone_	FU_Carina_
Spinal cord	*D*_max_	50.5 Gy	17	17	17	0	0	0	0
Esophagus	*D*_mean_	34 Gy	12	12	10	0	0	0	2
Heart	*V*_40_	50%	17	16	16	0	0	1	1
Heart	*V*_60_	30%	17	17	17	0	0	0	0
Lungs	*D*_mean_	20 Gy	7	10	10	3	3	0	0
Lungs	*V*_20_	30%	6	5	7	0	1	1	0
Lungs	*V*_30_	20%	4	3	3	0	0	1	1
Lungs-CTV	*D*_mean_	20 Gy	15	12	12	0	0	3	3
Lungs-CTV	*V*_20_	30%	12	10	11	1	1	3	2
Lungs-CTV	*V*_30_	20%	7	7	7	1	1	1	1
Lungs-GTV	*D*_mean_	20 Gy	9	10	10	1	1	0	0
Lungs-GTV	*V*_20_	30%	7	6	8	0	1	1	0
Lungs-GTV	*V*_30_	20%	4	3	3	0	0	1	1

Note: Dosimetric constraints used to evaluate dose changes are shown along with the number of plans out of eighteen total meeting each constraint in baseline, bone-aligned followup, and carina-aligned followup. Volume constraints are given in units of % structure volume. A constraint is defined as Improved if its limit was unmet in baseline and became met in followup, whereas Worsened signifies a constraint which was met in baseline but was unmet in followup.

**FIG. 6.**
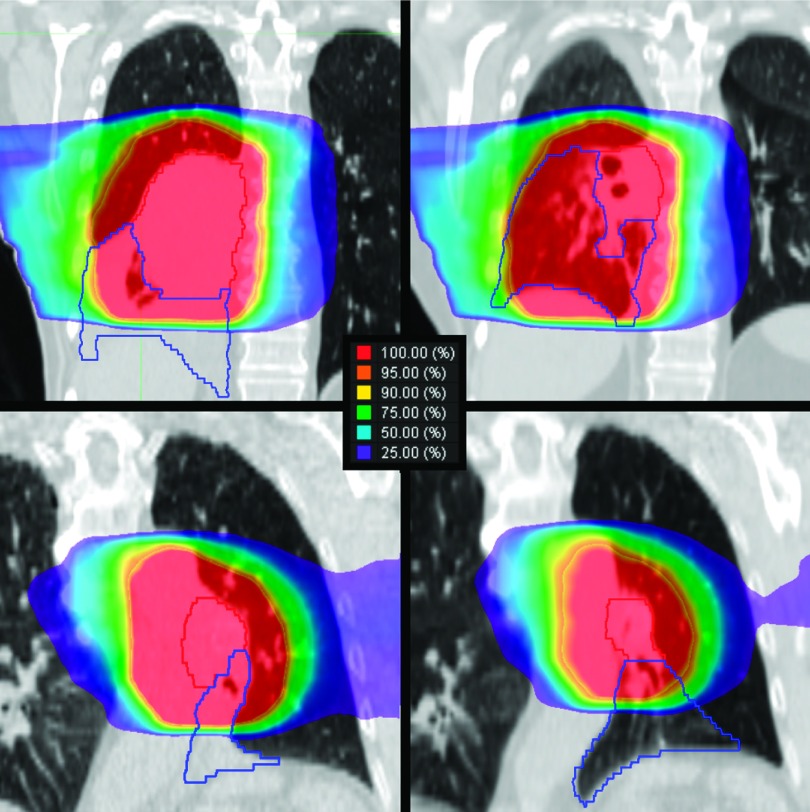
Dose distributions for two subjects, patient 5 (top) and patient 12 (bottom), are shown for baseline (left) and bone-aligned followup (right). Contours are shown for the lobe experiencing atelectasis resolution (blue) and the GTV (red). Dose is displayed as a percentage of prescription dose, 66 Gy for patient 5 and 58 Gy for patient 12. While both subjects experienced full resolution of whole lobe collapse, patient 5 had large dose changes while patient 12 showed modest differences due to the majority of atelectasis being a greater distance from the high dose region.

Figure 6 illustrates how atelectasis resolution had varying impact on changes seen in lung dose metrics. For patient 5, full resolution of atelectasis occurred which, in combination with significant tumor regression, caused the lung to expand and normal tissue to fall into the high dose region near the target. This resulted in increased dose to healthy lung and changes in lung metrics from baseline. Patient 12 also experienced full resolution of atelectasis. In this case, however, the atelectatic tissue was located mostly outside the high dose region and thus did not cause a large increase in healthy tissue dose. For reference, atelectatic lobe mass and density changes were, respectively, −29.4% and −59.9% for patient 5 and −39.1% and −64.7% for patient 12.

### 3.E. GTV coverage

Changes in GTV coverage were investigated in addition to OAR dose changes. Dose variation to GTV was analyzed rather than CTV, as it is not clear if the midtreatment CTV should be constructed by re-expansion of the GTV or by tracking the movement of tissue within the CTV region.^9,10^ Means (stdev) of changes in volume of GTV receiving at least the prescription dose, *V_Rx_*, were −5.5% (13.5%) and −5.3% (15.5%) for bone and carina alignments, respectively, and are reported in units of % GTV volume. *V_Rx_* changes ranged from −44.31% to +6.6% for bone alignment and from −57.3% to +10.3% for carina alignment. Due to the variation in prescription dose among patients of this study, the following dose changes are reported in units of % prescription dose. The mean (stdev) change in maximum GTV dose was 2.5% (4.2%) and ranged from −2.9% to +13.9% for bone alignment and was 2.3% (3.8%) and ranged from −2.1% to +13.3% for carina alignment. Similarly, mean (stdev) change in minimum GTV dose was 0.8% (8.9%) and ranged from −19.3% to +19.0% for bone alignment and was 0.2% (9.0%) and ranged from −17.1% to +19.5% for carina alignment. Tumor regression typically occurs during radiotherapy and can have an impact on target coverage. For reference, the mean (stdev) of GTV volume reduction at the time of the midtreatment imaging was 39.2% (26.7%) for the patients of this study.

## 4. DISCUSSION

This work has investigated mass, density, and OAR dose changes in radiotherapy subjects with obstructive lobar atelectasis using baseline and midtreatment fan-beam CT scans.

For a subset of patients, some dose constraints were unmet by the baseline plan. As described in Sec. 3.D, the large number of unmet baseline lung constraints was likely due to the difference in lung delineations between this study, which included atelectatic tissue as part of the lung, and clinical treatment planning, where collapsed lung is excluded. Five patients exceeded the mean esophagus dose limit in their baseline plans, though this was not surprising as the esophagus dose limit is a soft constraint which is sometimes exceeded when necessary to obtain adequate target coverage.

Predicting dosimetric change for a particular patient is challenging due to the multifactorial nature including amount and location of atelectasis at baseline, amount and shape of atelectasis resolution, location of organs at risk relative to target position, etc. For example, the largest of all dose changes occurred in a patient with partial resolution (mean esophagus dose increase >7 Gy), due to location of the esophagus relative to high dose gradients. Thus, the goal of the dosimetric portion of this study was to investigate the range and magnitude of such changes, on average, rather than try to predict the amount of dosimetric change for particular groups.

Midtreatment alignment based on carina, rather than bone, in the presence of atelectasis resolution causes less dose differences and slightly more dose constraints to improve during followup compared to their baseline status. Yet in both cases, while not always causing metrics to exceed clinical limits, large max and min differences from baseline occurred in lung dose/volume metrics. Heart *V*_40_ showed a maximum increase of over 9% for bone alignment and was only slightly reduced to 6% when the subject was aligned via carina.

While dose changes from baseline to followup were small when averaged across patients, a few subjects experienced large dose increases to critical structures from the intended dose/volumes of the baseline plan. Maximum dose to spinal cord varied substantially with a standard deviation across patients of 3 Gy for bone alignment. Likewise, mean dose to the esophagus had large variations across patients, with standard deviations of about 2.7 Gy for both alignment methods. Mean lung dose was expected to be relatively insensitive to changes in dose distribution yet increased in some subjects by over 4–7 Gy, depending on the lung definition. A high percentage of constraints were violated, and these unmet limits were spread over all patients rather than just a small subset, highlighting the need for adaptive planning.

Lung tissue was defined in three ways for the purposes of constraint evaluation. Inclusion of all lung tissue and tumor within the lung borders minimized the effects of tumor regression. Removal of the GTV from lungs assessed dose to tissue appearing as healthy lung in a CT image. Defining the lungs in this way included tissue adjacent to the tumor volume, which received high dose. RTOG 0617 evaluates lung constraints on the lungs minus CTV. By doing so, dose intended to be delivered to microscopic disease extension was excluded, leaving only healthy lung tissue where no dose was desired.

The constraint metric changes reported in Table I were consistent with the various lung definitions. Lung-GTV had the largest changes, reflective of the tumor regression which had occurred between baseline and followup. As the tumor shrunk, an increased volume of lung tissue was included in the high-dose region increasing the difference from baseline lung metrics. The smallest differences between baseline and followup resulted from the lung and lung-CTV definitions since these included parenchyma that were at a greater distance from the high dose region.

Relative mass and density changes were calculated on a lobe-by-lobe basis in order to reduce delineation uncertainty, as lobe fissures were more easily discerned. Healthy lobes and lobes which showed expansion or no change in atelectasis did not have significant mass change. Lobes with partial resolution of atelectasis by the midtreatment time point showed, on average, a decrease in mass. When full resolution of atelectasis occurred, mixed results were observed where some patients had increase in mass and others had decrease in mass, yet the mass change on average showed only a slight decrease of 2.8%. Lack of clarity in results for full resolution lobes is likely a consequence of limited numbers experiencing complete resolution of atelectasis (*n* = 4 for full resolution).

Given that resolution of atelectasis occurs through reaeration of collapsed lung tissue, we hypothesized that no mass change would occur during atelectasis resolution. Partial resolution results reported here demonstrate that there is reduction in overall lobe mass as the lobe transitions from a consolidated collapsed state back to healthy parenchyma. One possible explanation for decrease in mass is the additional presence of edema and/or infiltrate in atelectatic lung, which may resolve following re-aeration. Density change results were in alignment with expectations. Healthy lobes and those experiencing no change in atelectasis showed no significant changes in tissue density. Atelectatic lobes had decreases in density proportional to the degree of re-aeration, with full resolution lobes showing larger density decreases than partially resolving lobes. Although not statistically significant, median mass and density changes for healthy lobes were less than zero and warrant further investigation. Calculation of the cumulative dose requires deformable image registration which is challenged by large geometric changes, particularly when accompanied by mass and density variations such as those observed in this study. Understanding mass and density changes of atelectasis can help design new registration algorithms geared more toward accurate modeling and registration of these changes.

Previous studies have investigated the impact of atelectasis resolution on dose but with conflicting conclusions. One study implementing a traffic light protocol system to catch large setup errors on a perfraction basis found 6% of lung cancer patients experienced atelectasis resolution; all were flagged by the protocol, and 75% were severe enough to require inspection by an oncologist.^6^ Another investigation into dosimetric impact of atelectasis resolution and tumor regression found both changes to have minimal effect on dose yet stated that replanning may be necessary.^11^ However, their study only investigated tissue density changes, ignoring geometry changes, which likely explains the difference in results compared to the current work. Additionally, a study into the benefits of adaptive radiotherapy estimated that 30% of their subjects with atelectasis would gain no benefit from plan adaptation.^3^

Despite the differences in conclusions, the degree of atelectasis resolution among patient cohorts was similar to previous inquiries. Møller^3^ reported 110 ml median atelectasis volume change, whereas the subjects in this study had average and median atelectasis volume reductions of 144 ml and 73 ml, respectively. The study by Grams *et al.*^11^ simulated atelectasis resolution by replacing atelectatic tissue with intensities of lung parenchyma to create pseudo CT images on which dose was evaluated. The authors acknowledge that this would not realistically simulate re-aeration as such changes are usually accompanied by surrounding tissue deformation. Our study addressed this limitation by calculating dose on actual midtreatment images. Not only were tissue heterogeneities taken into account, but also real tissue deformations and displacements were present. The analysis by Møller *et al.*^3^ also relied on pseudo CT images for dose investigation and did not evaluate dose metrics for OARs. By using clinically relevant dose/volume metrics, the critical structure dose investigation of the current study provided an accurate assessment of the dosimetric impact of atelectasis resolution.

The dose change analysis presented in this study assumes the observed tissue changes occur prior to the start of treatment and remain through the entire treatment course. This dose calculation method provides a worst-case scenario of dose change and overestimates dose differences when atelectasis resolution occurs later in the treatment course. A more precise measure of dosimetric change would involve deformable registration of more frequent imaging and accumulation of the dose; however, this is challenging due to the inaccuracy of registration in presence of large changes. Random interfraction variations may reduce and/or increase dosimetric changes, and their combined effect may wash out over the full duration of treatment. A major limitation of the study was small patient number due to the limited number of NSCLC patients presenting with atelectasis and having repeat CT scans. Contour delineation variability, another important concern, was minimized by delineation of lung lobes rather than the atelectasis directly and through the use of a single observer when possible. Lobe fissures are relatively easy to identify, whereas determining correspondences between subvolumes of a lobe is challenging at best and becomes nearly impossible when partial lobar atelectasis is present. While several individuals provided assistance with organ delineation, a single, experienced radiation oncologist reviewed all contours and altered them when necessary. The small deviations of mass and density changes across repeat image pairs suggest delineation variability had a negligible impact on our results. Differentiating tumor from atelectasis was also challenging for a subset of patients, as little to no contrast differences were visible in the CT scans. Clinical GTV contours, which utilized positron emission tomography to assist in GTV delineation, were used as a guide to minimize this uncertainty.

## 5. CONCLUSIONS

Mass and density of lung parenchyma appeared to decrease on average by midtreatment regardless of the degree to which atelectasis resolved. Re-aeration of collapsed lung had an impact on normal tissue dose due to mass and density changes, with up to 44% of patients having 5 Gy/5% or larger variations in at least one clinical dose constraint metric.

## CONFLICT OF INTEREST DISCLOSURE

Elisabeth Weiss receives research support from Philips Healthcare and the National Institutes of Health, has a licensing agreement with Varian Medical Systems, and receives royalties from UpToDate. Gary Christensen maintains research grants from National Institutes of Health and has received a gift from Roger Koch to support research. Geoffrey Hugo receives research support from Philips Healthcare and the National Institutes of Health and has a licensing agreement with Varian Medical Systems.
